# Use of the i2b2 research query tool to conduct a matched case–control clinical research study: advantages, disadvantages and methodological considerations

**DOI:** 10.1186/1471-2288-14-16

**Published:** 2014-01-30

**Authors:** Emilie K Johnson, Sarabeth Broder-Fingert, Pornthep Tanpowpong, Jonathan Bickel, Jenifer R Lightdale, Caleb P Nelson

**Affiliations:** 1 Department of Urology, Boston Children’s Hospital, 300 Longwood Ave, HU 3rd Floor, Boston, MA 02115, USA; 2 Harvard-Wide Pediatric Health Services Research Fellowship, Boston, MA, USA; 3 Department of Pediatrics, Massachusetts General Hospital for Children, Boston, USA; 4 Department of Pediatrics, Ramathibodi Hospital, Mahidol University, Bangkok, Thailand; 5 Informatics Program, Boston Children’s Hospital, Boston, USA; 6 Information Systems Department, Boston Children’s Hospital, Boston, USA; 7 Division of Gastroenterology and Nutrition, Boston Children’s Hospital, Boston, USA

**Keywords:** Case–control studies, Methodology, Administrative data, Informatics

## Abstract

**Background:**

A major aim of the i2b2 (informatics for integrating biology and the bedside) clinical data informatics framework aims to create an efficient structure within which patients can be identified for clinical and translational research projects.

Our objective was to describe the respective roles of the i2b2 research query tool and the electronic medical record (EMR) in conducting a case-controlled clinical study at our institution.

**Methods:**

We analyzed the process of using i2b2 and the EMR together to generate a complete research database for a case–control study that sought to examine risk factors for kidney stones among gastrostomy tube (G-tube) fed children.

**Results:**

Our final case cohort consisted of 41/177 (23%) of potential cases initially identified by i2b2, who were matched with 80/486 (17%) of potential controls. Cases were 10 times more likely to be excluded for inaccurate coding regarding stones vs. inaccurate coding regarding G-tubes. A majority (67%) of cases were excluded due to not meeting clinical inclusion criteria, whereas a majority of control exclusions (72%) occurred due to inadequate clinical data necessary for study completion. Full dataset assembly required complementary information from i2b2 and the EMR.

**Conclusions:**

i2b2 was critical as a query analysis tool for patient identification in our case–control study. Patient identification via procedural coding appeared more accurate compared with diagnosis coding. Completion of our investigation required iterative interplay of i2b2 and the EMR to assemble the study cohort.

## Background

The i2b2 (informatics for integrating biology and the bedside) clinical data informatics framework was originally developed within the Partners Healthcare System, a large, integrated healthcare system based in Boston [1]. The first version of code for i2b2 was released publicly in 2007 with support from an NIH-funded National Center for Biomedical Computing [2]. Since its inception, over 200 scholarly articles have been published using data derived from i2b2 systems [3]. A major aim of i2b2 is to create a cost-effective and efficient way to identify patients for many types of clinical and translational research [1].

At Boston Children’s Hospital (BCH), the data contained within i2b2 is obtained from multiple administrative and electronic medical record data sources, including hospital and physician billing records and data generated over the course of clinical care. Specific available data types include demographics, medications, laboratory values, and *International Classification of Diseases, 9*^*th*^*Edition, Clinical Modification (ICD-9-CM*) billing and procedure codes. The clinical and administrative data sources feed into i2b2 to provide a searchable composite data repository that is used by clinical and translational researchers at our hospital. Clinical data becomes available to i2b2 as it becomes digitized, such that laboratory data is robust going back many years, whereas detailed prescription data has become more universally available as more providers have begun to use electronic, rather than paper, prescriptions. Therefore, availability of specific clinical data elements through i2b2 is largely time-dependent.

Our group at BCH recently used the i2b2’s ontological search framework to conduct a case–control study examining risk factors for the development of kidney stones among pediatric patients cared for at our institution who are fed via gastrostomy tube (G-tube) [4]. In order to assemble the database for this study, we undertook a multi-step process that required:

1. Access to the patient population of interest

2. Accurate identification of cases based on pre-specified inclusion and exclusion criteria

3. Assurance that cases arose from the same population from which we were drawing our matched controls

4. Case–control matching based on pre-specified matching criteria.

The objectives of this paper are to evaluate the role of i2b2 in creating the dataset for our case–control study, and to discuss the interplay between this research query tool and clinical data extracted through detailed chart review. In particular, we describe the utility of i2b2 for identifying cases and controls at a single institution, from our perspective as clinical investigators.

## Methods

**Figure 1:**
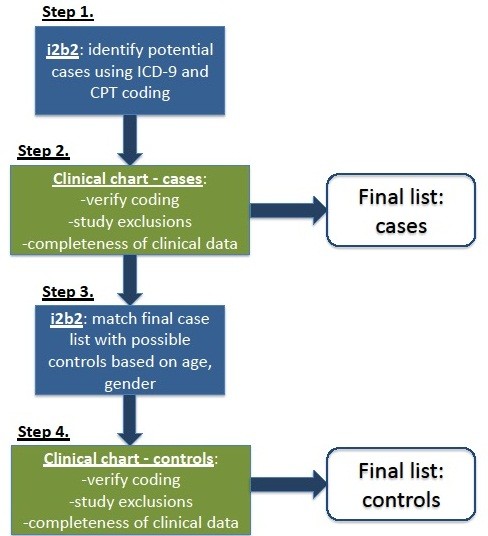
Strategy for assembly of case–control cohort – attached file.

A schematic of our data collection strategy is illustrated in Figure 1.

Potential cases (defined as patients with both G-tubes and kidney stones) were identified via an i2b2 query that requested the presence of at least 1 *ICD-9-CM* or *Current Procedural Terminology* (*CPT*) code for a G-tube (*ICD-9-CM* codes v44.1, 43.19, 43.11, 43.19, v55.1, 96.36, 97.02, 536.40, 536.41, 536.42, and 536.49; *CPT* codes 43246, 43653, 43750, 43760, 43830, 43831, 43832, 49440, 49450, 49465, and 74350) *and* at least 1 *ICD-9-CM* code representing a kidney stone (592.1, 592.2, 592.9). The initial query was conducted through the i2b2 workbench by our clinical research team, which produced a de-identified dataset. After IRB approval, the query was processed by the i2b2 Clinical Research Informatics Team, which allowed identification of potential cases.

The medical records of potential cases were then reviewed by the clinical research team to confirm eligibility for the study. Criteria for inclusion in the study were 1) an incident stone diagnosis between January 2005 and December 2011 and 2) a patient age of 1–21 at the time of stone diagnosis. Cases were excluded if 1) the presence of a G-tube or kidney stone could not be verified from within the clinical chart, 2) the kidney stone history predated G-tube placement, 3) there was insufficient data regarding tube feed formulation and/or regimen, or 4) the patient had received G-tube feeds for less than 3 months in the 12 months prior to the date of the kidney stone diagnosis. Cases were also later excluded if at least 1 eligible control could not be identified.

The final list of cases was then cross-referenced with patients identified via i2b2 as having one or more of the G-tube *ICD-9-CM* or *CPT* codes detailed above, in the absence of an *ICD-9-CM* code for a kidney stone, to identify potential controls. Criteria were then applied by the automated i2b2 system to match all potential control patients to the existing case list based on date of birth (±1 year) and gender.

The list of all potential controls was then sorted randomly by case number and sequentially examined via chart review until 2 controls were identified for each case. In addition to age and gender matching, each control was also matched to each case based on timing of G-tube placement. Specifically, to be eligible for the study, each control was required to have had a G-tube in place and to have been receiving tube feeds during at least 3 out of the 12 months prior to the date of kidney stone diagnosis for their matched case. Potential controls that did not meet these criteria were excluded, as were those in which the presence of a G-tube could not be verified or the nutritional data contained within their chart was deemed insufficient for the purposes of our study.

The reasons for exclusion of patients from the study cohort after initial inclusion via the i2b2 search algorithm were tabulated and categorized for both cases and controls. The study from which these data were derived was reviewed and approved by the Boston Children’s Hospital Institutional Review Board (Protocol # IRB-P000029420).

## Results

**Figure 2:**
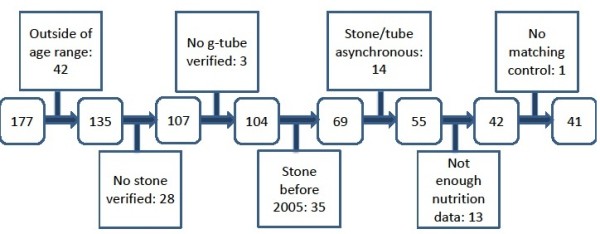
Detailed exclusions for case cohort (N = 41) – attached file.

The initial i2b2 query identified 177 potentially eligible cases as having both an *ICD-9-CM* and/or *CPT* code for a G-tube *and* an *ICD-9* code for a kidney stone. We excluded 136 cases due to aforementioned reasons (Figure 2). The final case cohort consisted of 41 cases (23.2% of the potential cases initially identified).

All identified cases (n = 41) were matched with 80 controls, who collectively represented 16.5% (80/486) of potential controls. All identified controls had both an *ICD-9-CM* and/or *CPT* code for a G-tube and fulfilled the matching criteria for at least one of the cases. There were 2 case patients with only 1 matching control and 1 potential case was excluded based on no eligible control. The mean number of potential control medical records reviewed in sequential order until a match for each case was identified was 9.9 +/- 8.4.

A comparison of the reasons for excluding cases vs. controls in the final analysis is illustrated in detail in Table 1. Among potential cases, 22.8% were excluded based on apparent inaccuracy of administrative codes, whereas this was true for only 7.6% of potential controls. Cases were 10 times more likely to be excluded for an inaccurate kidney stone code versus an inaccurate G-tube code. The majority of control exclusions (72.2%) occurred due to inadequate clinical (nutritional) data to complete our study.

**Table 1:** Reasons for exclusion of potential study participants – cases vs. controls

**Reason for exclusion**	**Excluded potential cases (N = 136)**	**Excluded potential controls (N = 406)**
**Inaccurate query**		
No kidney stone	28 (20.6)	NA
No G-tube	3 (2.2)	31 (7.6)
**Did not meet inclusion criteria**		
Outside of study age range	42 (30.9)	NA
Outside of study time period	35 (25.7)	NA
Kidney stone/G-tube asynchronous	14 (10.3)	82 (20.2)
**Inadequate clinical data**	13 (9.6)	293 (72.2)
**Other**		
No matching control	1 (0.73)	NA

## Discussion

In our study, i2b2 was critical as a research query tool for screening potential cases and controls as well as for conducting the matching query for our study. The full assembly of our case–control cohort required an iterative approach with multiple points of comparison between i2b2 and the clinical patient records. Approximately 23% of patients identified as eligible for our study based on the initial i2b2 query were eventually included as cases, and an average of 10 controls per case had to undergo chart review in order to attain a 2:1 match. Among potential cases that were ultimately excluded after chart review, 67% were excluded for not meeting our study inclusion criteria and 23% for the inability to verify the diagnosis of a stone or presence of a G-tube on an individual patient basis. In contrast, potential controls were more likely to be excluded for inadequate clinical data (72%), while coding inaccuracies were more rare (23% of potential cases vs. 8% of potential controls).

Throughout its design and implementation, we were acutely aware of potential methodological pitfalls to our case–control study [5,6]. Common limitations inherent to a case–control study design that applied to our investigation included 1) the need to explicitly define and apply the criteria for diagnosis of a case, 2) assurance that cases selected were incident cases, and 3) certainty that controls selected came from the same population as our cases [6]. However, given a paucity of prior investigations into both our primary study question, as well as our patient population of interest, we felt a case–control study design to be a reasonable first step. As such, we sought to be as standardized and comprehensive as possible in all aspects of our data collection process, starting with the cohort assembly.

Previous investigators have described the use of other databases sourced from billing/administrative tasks for conducting case–control studies. The proportion and magnitude of chart review necessary for each study appears to vary based on the specific diagnoses/conditions of interest for each investigation. In a manner similar to our use of i2b2, de Abajo and colleagues used a primary care research database to identify patients with a condition of interest based on administrative coding, and then conducted chart review based on the initial query [7]. In their study, the investigators initially identified cases with a diagnosis of upper gastrointestinal (UGI) bleed based on International Classification of Primary Care Code or free text related to UGI bleed, which was screened via natural language processing. Of the potential cases initially identified, 21% were included in the final case cohort after detailed chart review, similar to our final case inclusion rate of 23%. In a British case–control study examining whether routine primary care data could be used to identify children at risk of entering public care, Simkiss and colleagues were able to conduct both cohort assembly and data analysis without the use of detailed chart review [8]. The investigators did selectively validate the case selection strategy using clinician questionnaires. They were able to confirm case status in 93%, suggesting that an automated case selection algorithm for their condition of interest (placement of a child into public care) was a reasonable strategy. However, our experience and that of de Abajo and colleagues suggest that a completely automated case selection strategy is unlikely to yield accurate results, and in turn may require chart review to refine study cohorts.

One alternative to using a multi-source research database to facilitate the completion of institutional or multi-institutional case–control studies is the use of a registry-based strategy. Recent, expanded applications of data registries harness the benefits of open data sharing and advanced query and analytic tools in a similar fashion to the i2b2 framework. In one instance, the International Collaborative Gaucher Group Registry was able to establish a large, multi-institutional registry for a rare medical condition [9]. In this project, researchers and clinicians collaborated and agreed to enter specified data points on for each patient on a prospective basis, which then allowed for stratification of patients by several relevant clinical and demographic factors, which in turn facilitated investigation of risk factors for primary outcomes, such as avascular necrosis, in this rare-disease population. Such an approach is attractive for a variety of specific conditions. However, it may be unrealistic to expect multicenter groups to develop registries for every possible interesting combination of conditions (e.g. G-tube feeding plus kidney stone). Nevertheless, this illustration of using a centralized registry does argue for having complete lists of patients with particular conditions whenever feasible.

Certainly, an important consideration in using a research query tool that draws data from administrative billing records to conduct a case–control study is the potential to encounter inaccuracies in procedural and medical diagnostic coding key to identifying particular patient populations. In the clinical setting, several studies in prevalent diseases have used *ICD-9-CM* for case identification [10-13]. However, relying on *ICD-9-CM* codes alone may result in bias and disease misclassification. Recent studies have also supported the combined use of medical record review with an administrative database to validate the diagnosis [14,15]. For example, the use of both procedural (i.e., upper gastrointestinal endoscopy) and diagnostic (i.e., celiac disease) codes can improve the precision of case identification, as compared to the use of a diagnostic code alone [10]. Overall, procedural codes may have higher sensitivity and specificity compared with diagnosis codes [16,17]. In our investigation, cases were 10 times more likely to be excluded for an inaccurate kidney stone code versus an inaccurate gastrostomy tube code, likely reflecting that fact that our G-tube definitions used a combination of diagnostic and procedural codes, while the kidney stone definition relied on diagnostic codes alone.

Although our study required manual chart review to conform to the clinical constraints of our inclusion criteria, the i2b2 framework provided multiple important advantages that warrant mention. Most simply, i2b2 has both *ICD-9-CM* and *CPT* coding available for query, a feature that is not universal in administrative data sources. A specific advantage of i2b2 compared with traditional billing data is the ability to cross-reference several billing codes to generate a list of unique patients with multiple conditions of interest. i2b2 also contains an intuitive user interface that allows blinded (de-identified) screening queries to be conducted by the clinical research team based on *ICD-9-CM* and/or *CPT* coding. This feature allows clinical researchers to assess study feasibility prior to initiating a complete investigational protocol, or to obtaining formal IRB approval. Additional functionality of i2b2 included its ability to specify matched case–control groups based on predetermined criteria; in turn, i2b2 provided a critical means for our cases and controls to be selected from the same source population. Finally, i2b2 contains clinical data including laboratory results and biometric measurements, which could all be extracted into our dataset in a substantially streamlined, reliable fashion when compared with the alternative of manual chart abstraction.

At its current level of functionality, i2b2 did have several limitations. For instance, we were unable to comprehensively query clinical data beyond diagnosis and procedural coding. Even with the recent incorporation of natural language processing into i2b2, [18] it is difficult to imagine that the entire case–control cohort assembly process could be completed automatically without significant restructuring of the format of clinical data inputs. In the absence of clinical data restructuring, we could have potentially improved our initial query strategy by applying a more specific initial filter that included dates of service and/or by requiring particular encounter types. As an example, the majority of nutrition data were contained within certain visit types, so we could have required that a patient have a GI, complex care, nutrition or inpatient encounter to be eligible for further screening. We also considered limiting age ranges during the initial query so that fewer patients would be excluded due to being outside of the age range for our study. However, we were concerned that we could have missed potential cases or controls with more specific initial filters, so we elected to be broader in our search strategy for this index study using the i2b2 framework for case-cohort assembly.

## Conclusions

In summary, the i2b2 multi-source data informatics framework was critical as a query analysis tool for patient identification in our case–control study examining risk factors for kidney stones among G-tube fed children. Patient identification via procedural and diagnostic coding appeared more accurate compared with diagnostic coding alone. i2b2 also streamlined multiple additional aspects of our investigation, including the assessment of feasibility, case–control matching, and clinical data abstraction. Full completion of our study required an iterative interplay between administrative data and the more granular clinical record. Nevertheless, i2b2 remained a critical component at multiple steps in our study execution. Moving forward, enhancements such as natural language processing will continue to improve the i2b2 framework as a tool for clinical researchers, and will further distinguish this as a tool for generating clinical research datasets as compared with standard administrative data sources. In turn, we anticipate that its utility to case–control studies and other types of clinical investigations will continue to grow.

### Consent

This study was conducted as part of an investigation that was approved by the Institutional Review Board at Boston Children's Hospital. The study was reviewed and determined to be exempt from individual patient consent due to its retrospective nature.

## Abbreviations

i2b2: Informatics for integrating biology and the bedside; EMR: Electronic medical record; G-tube: Gastrostomy tube; BCH: Boston Children’s Hospital; ICD-9-CM: International classification of diseases, 9^th^ edition, clinical modification; CPT: Current procedural terminology; UGI: Upper gastrointestinal.

## Competing interests

The authors declare that they have no competing interests.

## Authors’ contributions

EJ conceived and designed the paper concept and analysis, drafted and revised the manuscript and approved the final version. SB contributed to the paper concept, assisted with drafting and revising the manuscript and approved the final version. PT contributed to the paper concept, assisted with drafting and revising the manuscript and approved the final version. JP contributed to the paper concept, assisted with drafting and revising the manuscript and approved the final version. JRL contributed to the paper concept, assisted with drafting and revising the manuscript and approved the final version. CPN contributed to the paper concept and analysis, assisted with drafting and revising the manuscript and approved the final version.

## Pre-publication history

The pre-publication history for this paper can be accessed here:

http://www.biomedcentral.com/1471-2288/14/16/prepub
